# Mr. Besant's New Book

**Published:** 1887-11-26

**Authors:** 


### MR. BESANT'S NEW BOOK.

Mr. Besant's contribution to the yearly avalanche of Christmas books "Katharine Regina,"* is one of those philanthropic studies of existing social conditions which we have come to expect from him, and which have undeniably enhanced his reputation, though they have tended to spoil his art. In his new story Mr. Besant takes up the cause of his favourite heroines, the working women, but they are of a new grade—those unfortunate gentlewomen who earn a scanty living as daily governesses, copyists, type-writers, translators, and sometimes aspire to be authors and painters. If they are fortunate they can live in a gloomy boarding-house, whose threshold no male foot is allowed to cross, at the rate of fifteen-pence halfpenny a-day; if they are unfortunate, and get out of work, they must go to the workhouse or die of starvation. The personages of the story are all familiar characters—the orphan kept unjustly out of her fortune, the lover reported dead but, as we all know, sure to reappear at the end of the story in time to confute the wicked lawyer who has swindled his sweetheart: these are old friends of the reader of fiction. The story in itself contains nothing that is original, but the environment, on which the true interest depends, is altogether new to fiction. It is not a cheerful environment, and the story, in spite of Mr. Besant's familiar brightness of style, is decidedly a gloomy one; for though Katie Willoughby's lover comes back to crown her with wealth and happiness, the reader's mind cannot but dwell on the miserable, loveless, hopeless lives of the sisterhood whom she left in Harley House—a sad band to whom neither wealth nor happiness are likely to come. The author does indeed picture a time when an army of affectionate and chivalrous youths will rescue these Andromedas of our day from that awful monster, the law of supply and demand; but this, we fear, is one of Mr. Besant's millennial dreams. And, save by marriage, there is no escape from their misery. They can never rise to eminence, never even to respectable mediocrity, in the work they profess to do, for the narrator of their woes himself admits that they do not like their work and that they are incompetent to do it. What then can be done for them? Nothing. Pity and charity may modify the sufferings of individual members; but, as a class, the incompetent, whether gently born or not, are beyond relief. It is doubtful if even the matrimonial remedy is a good one, though it fulfils what Mr. Besant seems to think is the legitimate ambition of every woman—to sit idle and let a man pour into her lap the money he has earned. It does not follow that because a woman has failed in any other career she will succeed in the difficult, never-ending duties of wife and mother. And while our population statistics show that in this country women outnumber men by at least a million, marriage, as an escape from the necessity of labour, is a thing impossible to many. Many women must earn their living; neither justice nor philanthropy can alter that fact; and we cannot but think that a story-teller whose aim, like Mr. Besant's, is to instruct as well as to amuse, would do better, even for the objects of his sympathy, if he taught them to do the work that falls to them with whole-souled energy, rather than encourage them to whine at their fate, labouring in that half-hearted fashion which makes women's work so often inferior to men's, and is the real cause of their wages so often sinking to the starvation point, on the chance of some man—any man—relieving them of the necessity for work—surely a poor ideal of marriage! There will still be plenty of work for charity to do. Help given to the competent till they win recognition and reward is money well invested; help given to the incompetent is a casting of bread upon the waters, without the assurance that after many days it will return, or even permanently modify the condition of the waters that absorb it.

* "Katharine Regina." By Walter Besant, 1s. Bristol: Arrowsmith.

